# Use of a New Prevention Model in Acute Care Surgery: A Population Approach to Preventing Emergency Surgical Morbidity and Mortality

**DOI:** 10.1097/AS9.0000000000000188

**Published:** 2022-07-25

**Authors:** Gregory L. Peck, Shawna V. Hudson, Jason A. Roy, Vicente H. Gracias, Brian L. Strom

**Affiliations:** From the * Department of Surgery, Rutgers Robert Wood Johnson Medical School, Piscataway, NJ; † Department of Health Behavior, Society, and Policy, Rutgers School of Public Health, Piscataway, NJ; ‡ New Jersey Alliance for Clinical and Translational Science, New Brunswick, NJ; § Department of Family Practice, Rutgers Robert Wood Johnson Medical School, Piscataway, NJ; ∥ Rutgers Cancer Institute of New Jersey, New Brunswick, NJ; ¶ Department of Biostatistics and Epidemiology, Rutgers School of Public Health, Piscataway, NJ; # Rutgers Biomedical and Health Sciences, Newark, NJ.

**Keywords:** acute care surgery, epidemiology of emergency surgical disease, population health, primary, secondary, tertiary prevention

## MORBIDITY, COSTS, AND MORTALITY FROM EMERGENCY SURGICAL DISEASE

Patients’ risk of emergency surgical disease and death after being treated with an emergent general surgical procedure remains an ongoing public health burden in the United States.^1^ Emergency surgical disease accounts for 11% of all surgical admissions, with 7 operative procedures accounting for 80% of the total burden of disease.^2^ The association of emergency surgery with high healthcare utilization and morbidity, including preventable postoperative complications and a $30 billion cost annually, suggests an opportunity to improve the value of surgical expenditures by shifting emergent to elective surgery or nonsurgical care.^3,4^

An even more concerning issue is high mortality; emergency general surgical disease represents over 50% of all surgical mortality.^2^ This results, in part, from the surgeon’s sole focus on tertiary prevention, that is, preventing mortality and disability among those undergoing emergency surgery, leaving a gap in primary and secondary prevention that could assist in preventing surgical disease and its progression toward emergency in the first place.

## ACUTE CARE SURGERY AND PREVENTION OF EMERGENCY SURGICAL DISEASE

While most acute care surgeons will of course continue to focus on tertiary prevention, the field of acute care surgery (ACS) has a unique opportunity to activate primary and secondary prevention and shift a proportion of the total population away from the requisite tertiary prevention, making a larger impact on the health of the public and the value of care across the population as a whole. Empirical solutions to the significant morbidity and mortality from emergency surgical disease require more than improving surgical care at the hospital level and innovative technical tools for surgery. The immediate goals of an acute care surgeon to prevent morbidity and mortality from a strangulated hernia, perforated diverticulitis, or gangrenous gallbladder would benefit from the study of risk associated with, and from, what led up to disease and hospitalization being emergent. In trauma care, violence prevention that occurs in the community is more population centered than gaining proximal and distal control of a patient’s life-threatening hemorrhage in the operating room after massive transfusion. Rather than exhausting the resources we have on the front lines of hospital healthcare to resuscitate each individual vulnerable patient, the field of ACS may play a unique role with social and economic responsibility by also prioritizing preventive interventions distal from, but informed by, the limits of tertiary prevention.

By developing timely programs of multidisciplinary research at the intersection of epidemiology and emergency surgery, acute care surgeon scientists can participate more closely in the modification of biopsychosocial etiologic factors, combining large population data, community engagement, and social research. This is important because, as acute care surgeons striving to achieve patient centered care and optimal outcomes, we know that we are not the ones who should be revealing to a patient and loved ones a diagnosis of advanced colorectal cancer discovered acutely. We do not generally have a prior relationship with the patient and will not likely have a subsequent long-term relationship with them. This is the role of a primary care provider. Yet, as high as one in 6 patients with colon cancer may present to an acute care surgeon previously undiagnosed.^5^ Further, the disease is more likely to be advanced when discovered in this way. Individuals are 73% significantly less likely to have emergency hospital admission, obstruction, or perforation if they have up-to-date colorectal cancer screening (primary prevention), not to mention also with polypectomy, that is, secondary prevention.^6^ An estimated 16% of the US acute care surgeons’ responsibility can be soft tissue infections and necrosis requiring extremely morbid, disfiguring, and functional debilitating operations, that are also potentially preventable through better control or prevention of prior diabetes.^1^ Further, if society had better control of the intravenous opioid epidemic in the United States, this would also markedly decrease the need for this surgery.

**FIGURE 1.**
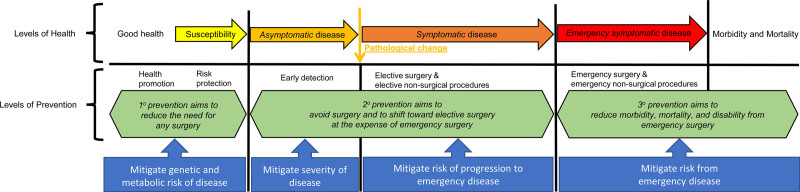
Primary, secondary, and tertiary prevention of emergency surgical disease and emergency surgery morbidity and mortality.

Thus, we propose a model of primary and secondary prevention to shift away from tertiary prevention as a step toward more permanent integrated solutions within the field of ACS (Fig. 1). An imbalance among primary, secondary, and tertiary prevention in the epidemiology of emergency surgical disease is evident when emergent forms of surgery are nearly as common as elective forms.^7^ The model is informed by the 3 following principles in the epidemiology of emergency surgical disease.

1. Elective surgery required for symptomatic disease is preventable.
2. Urgent surgery required for nonemergency-nonelective symptomatic disease is preventable.
3. Emergency surgery required for emergency symptomatic disease is preventable.

This broader preventive construct is important to circumvent the bias in the current literature, where the total population is usually not the perspective used.

## PRIMARY PREVENTION

Primary prevention of emergency surgery includes efforts to reduce the population’s disease in general, for example, ensuring access to healthy foods and physical environment over the lifespan. This may not be an equitable solution when the most vulnerable live in challenging environments with food deserts or a lack of primary care. Rather, it needs to be the responsibility of a health care system, which lacks prevention priorities currently, and policy change is a potential, but largely unproven, change lever. Although counseling of vulnerable patients on diet and lifestyle should occur as a responsibility of primary care clinicians in the education of their patients, this is difficult if patients depend for their primary care on the emergency room. Instead, grassroots community engagement can be woven into productive alternate payment models whereby the collective financial interest of health systems, payers, and surgeons is linked to the outcomes of the targeted population. For treatment of gallstone disease, for example, the progression from good health to asymptomatic disease, the actual formation of gallstones, introduces an opportunity for a prevention effort seemingly further removed from the current field of ACS. But, could diet changes, or treatment of hyperlipidemia with statins, prevent the eventual need for cholecystectomy?^8^ While some may conclude it is not the responsibility of the emergency surgeon, whose job is to treat his/her/their patient, to ask such questions, we propose that we have a responsibility as a field to minimize invasive interfaces with the population. This would be analogous to what Dentistry did with fluoridation, and what ACS is attempting with prevention of gun violence. After all, advanced surgical disease is not a direct reflection of patient’s independent decision-making but depends on the degree they have been educated about healthy behavior, limited by the structural realities suffered.

## SECONDARY PREVENTION

Secondary prevention of emergencies aims to reduce morbidity and mortality from the progression of asymptomatic to symptomatic and then emergent disease. One approach is to identify the heterogeneity of intergenerational effects of epigenetic, biologic, and environmental mechanisms on disease progression.^9^ For example, exposure to toxins in utero and genetic cholesterol biosynthesis, as well as metabolomics, and water quality, may offer new evidence related to prevention of asymptomatic to symptomatic gallstone disease.

Lack of primary care has also been found to be associated with emergency versus elective cholecystectomy.^10^ When combining inpatient and outpatient care of symptomatic gallstone disease, cholecystectomy is the most performed abdominal surgery in the United States, with inpatient and outpatient cases nearly equal in number.^11^ This is concerning considering a fivefold increase in mortality found in one state’s experience when the surgery is done emergently versus electively.^12^ An estimate of the potential number of deaths each year in the United States from intervention of symptomatic gallstone disease (not just cholecystectomy) is not insignificant.

If nearly 30 million Americans have risk of developing symptomatic gallstone disease, and 8% of those have symptomatic gallstone disease requiring tertiary prevention, and 1% die (conservative estimate across nonsurgical procedures and elective, nonemergency-nonelective, and emergency cholecystectomy), that is 24,000 potentially avoidable deaths each year. Indeed, gallstone disease is a useful use-case as the number of deaths remains high, it is preventable mortality, we know already that too much is emergent, and much of the disease’s natural history is known. But evidence does not explain the mechanisms or etiologies in the contemporaneous era perpetuating this preventable mortality.

The high volume and mortality of emergency general surgery call for the integration of emergency room, inpatient, outpatient, and physical environmental data to better achieve health equity in the total population. For example, could the population discharged from the emergency room be enrolled in “risk-stratification of emergency gallstone disease” programs that incentivize prevention?^13^ Could we take the next steps in addressing how and why Black patients are found to have lower odds of having ambulatory versus inpatient cholecystectomy and higher emergency postoperative mortality than White patients?^14,15^ Can we explore also how household income and ability to purchase preventive medications is also directly related to progression of digestive disease requiring the emergency surgeon? Never should peptic ulcer disease, or other gastrointestinal diseases like diverticular or inflammatory bowel disease, require laparotomy for perforation, bleeding, or obstruction because a patient was not able to pay for primary or secondary prevention, for example, of medically treatable disease.

Further, could changes in policy or payer contracts address lack of healthcare coverage for populations who would benefit from this preventive and elective care? The challenge is patients who have trouble presenting for elective surgery because its reimbursement is under-reimbursed by a health insurance system that fully covers emergency services. Underinsured populations have a safety-net workaround—emergency rooms—but this leads to a larger problem—a lack of prevention. Federal policy has already stimulated health insurers in the United States to cover certain preventive care without requiring a patient to pay a deductible, copayment, or coinsurance. An analogous option would be expansion of currently covered elective services considered preventive of emergency surgical disease, perhaps as simple as an ambulatory versus inpatient version of laparoscopic cholecystectomy, given the latter has been shown to have more value, less cost, and lower perioperative readmission.^16^

Fortunately, the national evolution toward population health and value-based care may offer financial incentives to making primary and secondary prevention of emergency surgical disease happen. For example, an estimated $1 billion over 10 years could be saved from a shift toward 3 elective procedures nationally.^17^ Does increasing access to accredited free-standing ambulatory surgical centers as part of the alternative payment models expedite appropriate elective surgical care, while decreasing preventable hospital mortality? If data prove this correct, then true population health reimbursements will hopefully drive it. And, in the meantime, insurers will be informed to change their reimbursement policies to encourage it, to first prevent suffering and loss of life, but also to save money.

## FINAL COMMENTS

Future achievement in surgical healthcare may require that we cultivate the larger preventive ecosystem rather than ballooning the investment in emergency healthcare, a symptom of, not a solution to, current inequities. Efforts in changing the types of services that alter the amount of tertiary prevention can be thought of as “damage control” applied in the community. One might suggest a place for initial action is increasing access to outpatient elective healthcare for underrepresented groups with barriers to preventive care, for example, by increasing health insurance. A next step we recommend is a focus on diseases currently requiring as much emergency as elective care as a tangible pilot, for example, gallstone disease and cholecystectomy. The practice and study of emergency surgery then has the potential to broaden from a focus on the patient in the emergency and hospital operating rooms to the patient who no longer requires either, whose emergency surgical disease is prevented. By focusing upstream from diagnoses and treatments that occur emergently, the field of ACS may strive to achieve a novel population health approach by seeking to prevent the most deaths from exposure to emergency surgery. This is a shift from the reactive treatments of emergency disease to proactive prevention of it, proven by a decrease in asymptomatic, symptomatic, and emergency symptomatic epidemiology. Understanding the trends in the risk of disease requiring emergency surgery for the total population, then determining multidimensional ways of treating it, and more importantly, preventing it, will evolve the field and its broader impact.

## ACKNOWLEDGMENTS

G.L.P. participated sufficiently in the intellectual content and writing of the article to take public responsibility for it. He has reviewed the article, believe it represents valid work, and approves it for submission. S.V.H. participated sufficiently in the intellectual content and writing of the article to take public responsibility for it. She has reviewed the article, believe it represents valid work, and approves it for submission. J.A.R. participated sufficiently in the intellectual content and writing of the article to take public responsibility for it. He has reviewed the article, believe it represents valid work, and approves it for submission. V.H.G. participated sufficiently in the intellectual content and writing of the article to take public responsibility for it. He has reviewed the article, believe it represents valid work, and approves it for submission. B.L.S. participated sufficiently in the intellectual content and writing of the article to take public responsibility for it. He has reviewed the article, believe it represents valid work, and approves it for submission.

## References

[1] Gale SC Shafi S Dombrovskiy VY. The public health burden of emergency general surgery in the United States: a 10-year analysis of the Nationwide Inpatient Sample--2001 to 2010. J Trauma Acute Care Surg. 2014;77:202 208. 25058242 10.1097/TA.0000000000000362

[2] Scott JW Olufajo OA Brat GA. Use of national burden to define operative emergency general surgery. JAMA Surg. 2016;151:e160480. 27120712 10.1001/jamasurg.2016.0480

[3] Ross SW Kuhlenschmidt KM Kubasiak JC. Association of the risk of a venous thromboembolic event in emergency vs elective general surgery. JAMA Surg. 2020;155:503 511. 32347908 10.1001/jamasurg.2020.0433 PMC7191471

[4] Ogola GO Gale SC Haider A. The financial burden of emergency general surgery: national estimates 2010 to 2060. J Trauma Acute Care Surg. 2015;79:444 448. 26307879 10.1097/TA.0000000000000787

[5] Gunnarsson H Holm T Ekholm A. Emergency presentation of colon cancer is most frequent during summer. Colorectal Dis. 2011;13:663 668. 20345966 10.1111/j.1463-1318.2010.02270.x

[6] Decker KM Lambert P Nugent Z. Time trends in the diagnosis of colorectal cancer with obstruction, perforation, and emergency admission after the introduction of population-based organized screening. JAMA Netw Open. 2020;3:e205741. 32453385 10.1001/jamanetworkopen.2020.5741 PMC7251446

[7] Mullen MG Michaels AD Mehaffey JH. Risk associated with complications and mortality after urgent surgery vs elective and emergency surgery: implications for defining “Quality” and reporting outcomes for urgent surgery. JAMA Surg. 2017;152:768 774. 28492821 10.1001/jamasurg.2017.0918 PMC5710495

[8] Bodmer M Brauchli YB Krähenbühl S. Statin use and risk of gallstone disease followed by cholecystectomy. JAMA. 2009;302:2001 2007. 19903921 10.1001/jama.2009.1601

[9] Di Ciaula A Portincasa P. Recent advances in understanding and managing cholesterol gallstones. F1000Res. 2018;7:F1000. 10.12688/f1000research.15505.1 PMC6173119 30345010

[10] Moore AN Carmichael H Steward L. Cholecystectomy: exploring the interplay between access to care and emergent presentation. J Surg Res. 2019;244:352 357. 31323390 10.1016/j.jss.2019.06.070

[11] Steiner CA Karaca Z Moore BJ. Surgeries in hospital-based ambulatory surgery and hospital inpatient settings, 2014: statistical brief #223. In: Healthcare Cost and Utilization Project (HCUP) Statistical Briefs. Agency for Healthcare Research and Quality (US); 2017. Available at: www.hcup-us.ahrq.gov/reports/statbriefs/sb223-Ambulatory-Inpatient-Surgeries-2014.pdf. 28722845

[12] To KB Cherry-Bukowiec JR Englesbe MJ. Emergent versus elective cholecystectomy: conversion rates and outcomes. Surg Infect (Larchmt). 2013;14:512 519. 24274058 10.1089/sur.2012.160

[13] Williams TP Dimou FM Adhikari D. Hospital readmission after emergency room visit for cholelithiasis. J Surg Res. 2015;197:318 323. 25959838 10.1016/j.jss.2015.04.032 PMC4466203

[14] Janeway MG Sanchez SE Rosen AK. Disparities in utilization of ambulatory cholecystectomy: results from three states. J Surg Res. 2021;266:373 382. 34087621 10.1016/j.jss.2021.03.052

[15] Armenia SJ Pentakota SR Merchant AM. Socioeconomic factors and mortality in emergency general surgery: trends over a 20-year period. J Surg Res. 2017;212:178 186. 28550905 10.1016/j.jss.2017.01.015

[16] Friedlander DF Krimphove MJ Cole AP. Where is the value in ambulatory versus inpatient surgery? Ann Surg. 2021;273:909 916. 31460878 10.1097/SLA.0000000000003578

[17] Haider AH Obirieze A Velopulos CG. Incremental cost of emergency versus elective surgery. Ann Surg. 2015;262:260 266. 25521669 10.1097/SLA.0000000000001080

